# ***QuickStats*:** Number* **of Youths Aged 2–19 Years and Adults Aged ≥20 Years with Obesity****^†^** **or Severe Obesity****^§^** **— National Health and Nutrition Examination Survey, 2015–2016**

**DOI:** 10.15585/mmwr.mm6734a7

**Published:** 2018-08-31

**Figure:**
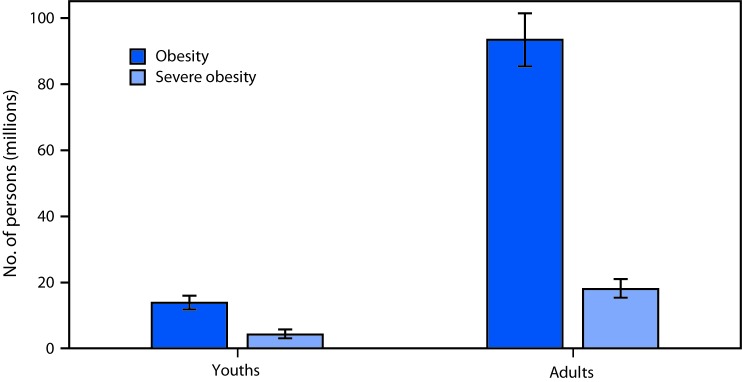
During 2015–2016, in the United States, there were 13.7 million youths (18.5%) with obesity, including 4.2 million youths (5.6%) with severe obesity. During this same period, in the United States, there were 93.3 million adults (39.8%) with obesity, including 17.8 million adults (7.6%) with severe obesity.

**Sources:** Hales et al. Trends in Obesity and Severe Obesity Prevalence in US Youth and Adults by Sex and Age, 2007–2008 to 2015–2016. https://jamanetwork.com/journals/jama/fullarticle/2676543; National Center for Health Statistics, National Health and Nutrition Examination Survey, 2015–2016. https://www.cdc.gov/nchs/nhanes.htm.

